# Critical literature review and pooled analysis of diagnostic accuracy of Ortho VITROS SARS-CoV-2 antigen test for diagnosing acute SARS-CoV-2 infections

**DOI:** 10.5937/jomb0-36107

**Published:** 2022-10-15

**Authors:** Giuseppe Lippi, Riccardo Nocini, Henry M. Brandon

**Affiliations:** 1 University of Verona, Section of Clinical Biochemistry and School of Medicine, Verona, Italy; 2 University of Verona, Department of Surgery, Dentistry, Paediatrics and Gynaecology, Unit of Otorhinolaryngology, Verona, Italy; 3 The Heart Institute, Cincinnati Children's Hospital Medical Center, Cardiac Intensive Care Unit, Cincinnati, OH, United States of America; 4 Texas Biomedical Research Institute, Disease Intervention & Prevention and Population Health Programs, San Antonio, TX, United States of America

**Keywords:** Sars-CoV-2, Covid-19, immunoassay, diagnosis, antigen, Sars-CoV-2, Covid-19, imunoesej, dijagnoza, antigen

## Abstract

**Background:**

The present study is aimed at reviewing and meta-analyzing the currently published data on the diagnostic accuracy of Ortho VITROS SARS-CoV-2 antigen test for diagnosing acute SARS-CoV-2 infections.

**Methods:**

An electronic search was conducted in Scopus and Medline with the keywords "VITROS" AND "antigen" AND "COVID-19" OR "SARS-CoV-2" AND "immunoassay" within the search fields "TITLE" AND "ABSTRACT" AND "KEYWORDS", without no date (i.e., up to January 23, 2022) or language restrictions, aimed at detecting documents reporting the diagnostic accuracy of this SARSCoV-2 immunoassay compared with reference molecular diagnostic methods.

**Results:**

Overall, 5 studies (n=2734 samples) were finally included in our pooled analysis, four of which also provided diagnostic sensitivity in oro-and nasopharyngeal samples with high viral load. The pooled cumulative diagnostic sensitivity and specificity were 0.82 (95%CI, 0.78-0.86) and 1.00 (95%CI, 1.00-1.00), respectively, whilst the area under the curve was 0.995 (95%CI, 0.993-0.997), the cumulative agreement 97.2% (95%CI, 96.5-97.8%), with 0.89 (95%CI, 0.86-0.91) kappa statistics, thus reflecting an almost perfect concordance with reference molecular biology techniques. The pooled diagnostic sensitivity in samples with high viral load was as high as 0.98 (95%CI, 0.96-0.99).

**Conclusions:**

These results confirm that the automated and high-throughput Ortho VITROS SARS-CoV-2 antigen test may represent a valuable surrogate of molecular testing for diagnosing acute SARS-CoV-2 infections, especially in subjects with high viral load.

## Abstract (sr)

**Uvod:**

Ova studija ima za cilj pregled i meta-analizu trenutno objavljenih podataka o dijagnostičkoj tačnosti Ortho VITROS SARS-CoV-2 antigen testa za dijagnostikovanje akutnih SARS-CoV-2 infekcija.

**Metode:**

Sprovedena je elektronska pretraga u Scopusu i Medline-u sa ključnim rečima "VITROS" i "antigen" i "COVID-19" ili "SARS-CoV-2" i "imunoassay" u okviru polja za pretragu "NASLOV" i "ABSTRACT" i "KLJUČNE REČI", bez datuma (tj. do 23. januara 2022.) ili jezičkih ograničenja, sa ciljem da se otkriju dokumenti koji izveštavaju o dijagnostičkoj tajnosti ovog imuno eseja SARS-CoV-2 u poređenju sa referentnim molekularnim dijagnostičkim metodama.

**Rezultati:**

Ukupno, 5 studija (n=2734 uzorka) je konačno uključeno u našu objedinjenu analizu, od kojih su četiri takođe pružile dijagnostiku osetljivost u oroi nazofaringealnim uzorcima sa visokim virusnim opterećenjem. Objedinjena kumulativna dijagnostika osetljivost i specifičnost bile su 0,82 (95%CI, 0,78-0,86) i 1,00 (95%CI, 1,00-1,00), respektivno, dok je površina ispod krive bila 0,995 (95%CI, 0,9973-0). kumulativno slaganje 97,2% (95%CI, 96,5-97,8%), sa 0,89 (95%CI, 0,86-0,91) kapa statistike, što odražava skoro savršenu saglasnost sa referentnim tehnikama molekularne biologije. Objedinjena dijagnostička osetljivost u uzorcima sa visokim virusnim opterećenjem bila je čak 0,98 (95% CI, 0,96-0,99).

**Zaključak:**

Ovi rezultati potvrđuju da automatizovani i visoko propusni Ortho VITROS SARS-CoV-2 antigen test može predstavljati vredan surogat molekularnog testiranja za dijagnostikovanje akutnih SARS-CoV-2 infekcija, posebno kod subjekata sa visokim virusnim opterećenjem.

## Introduction

Regardless of its still unclear origin [1], the ongoing coronavirus disease 2019 (COVID-19) pandemic caused by the severe acute respiratory syndrome coronavirus 2 (SARS-CoV-2) is dramatically disrupting the integrity and efficiency of most healthcare systems worldwide [2]. An already critical situation has recently become even worse boosted by the emergence of new SARS-CoV-2 lineages characterized by increased transmissibility, such as the Omicron (B.1.1.529) variant [3], which is now responsible for a dramatic surge of SARS-CoV-2 infections all around the world due to a magnified capability to escape the host immune response [4]. Such a tremendous increase of SARS-CoV-2 infections is causing a kaleidoscope of adverse consequences on clinical laboratories, many of which are no longer capable to withstand the pressure of performing enormous volumes of diagnostic tests with a suitable turnaround time. In keeping with this, a worldwide survey performed by the American Association of Clinical Chemistry (AACC) has evidenced that more than half of respondent laboratories declared that they were actually unable to purchase a sufficient number of tests to analyze all specimens received [5].

Although the reference approach for diagnosing an acute SARS-CoV-2 infection still encompasses the detection of viral RNA in a respiratory specimen by means of a nucleic acid amplification tests (NAATs), namely with (real-time) reverse transcription polymerase chain reaction (rRT-PCR) [6], these methods are essentially time-consuming, have an intrinsically low throughput and required dedicated instrumentation and skilled personnel [7], thus paving the way to development and commercialization of immunoassays aimed at detecting SARS-CoV-2 antigens rather than RNA. Besides rapid diagnostic test for SARS-CoV-2 antigen detection (RDT-Ag) [8], whose diagnostic performance are extremely heterogeneous and mostly unsuitable for replacing NAATs in most circumstances (i.e., the diagnostic sensitivity is typically low, around 60%) [9], laboratory-based, accurate and fully-automated SARS-CoV-2 antigen immunoassays have been proposed as possible solution to overcome the current testing backlog, shortage of supply and delayed generation of test results. Among the various methods, the novel Ortho VITROS SARS-CoV-2 antigen test is a chemiluminescent immunoassay that has been specifically developed for supporting the diagnosis of acute SARS-CoV-2 infections. To this end, the present study is aimed at reviewing and summarizing the currently published information on the diagnostic accuracy of this novel fully-automated and highthroughput technique. We present the following article in accordance with the PRISMA (Preferred Reporting Items for Systematic Reviews and Meta-Analyses) reporting checklist.

## Materials and methods

### Immunoassay description

The Ortho VITROS SARS-CoV-2 antigen test (Ortho-Clinical Diagnostics, Raritan, NJ, USA) is a chemiluminescent immunoassay that has been developed for detecting SARS-CoV-2 nucleocapsid (N) protein on fully-automated Ortho platforms (i.e., VITROS 3600 Immunodiagnostic System and VITROS 5600/XT 7600 Integrated Systems). The test been recently cleared by the US Food and Drug Administration (FDA) with Emergency Use Authorization (EUA). Briefly, the immunoassay involves a two-stage reaction. In the first step, the SARS-CoV-2 nucleocapsid (N) protein present in the test sample reacts with coated monoclonal anti-SARS-CoV-2 antibodies. After removing unbound material from the test reaction chamber by washing, anti-SARS-CoV-2 monoclonal antibodies labeled with horseradish peroxidase (HRP) are added to the reaction mixture. Unbound conjugate is eliminated by washing, whilst the HRPbound conjugate is measured with a luminescent reaction by adding a luminogenic substrate and an electron transfer agent to the reaction wells. The signal to cutoff ratio (S/C) proportionally increases with the amount of SARS-CoV-2 nucleocapsid antigen present in the test sample, with reactivity (i.e., diagnostic positivity) set at ≥1 S/C threshold. The sample volume is just 80 μL, the turnaround time of the test is around 48 min, whilst the throughput (depending on the automated platform) ranges between 120 and 150 samples per hour.

According to manufacturer's specifications, the limit of detection ranges between 5.0×10^2^ and 3.0×10^3^ Median Tissue Culture Infectious Dose (TCID_50_)/mL, the positive and negative percent agreement for diagnosing acute SARS-CoV-2 infections are as high as 80% (95% confidence interval [95%CI], 57-88%) and 100% (95%CI, 95-100%), respectively. Other pre-analytical and analytical characteristics are not presented here for space constrains, but are thoroughly described in the package insert [10].

### Search strategy

The search strategy used in this study is summarized in Table 1. Briefly, the electronic search was conducted in the two scientific databases Scopus and Medline (on PubMed interface) based on the keywords »VITROS« AND »antigen« AND »COVID-19« OR »SARS-CoV-2« AND »immunoassay« within the search fields »TITLE« AND »ABSTRACT« AND »KEYWORDS«, without no date (i.e., up to January 23, 2022) or language restrictions, aimed at detecting potential documents which reported the diagnostic accuracy of Ortho VITROS SARS-CoV-2 antigen test compared with reference molecular diagnostic methods. The two authors (G.L. and B.M.H.) assessed the title, abstract and full text (when available) of all items that could be detected based on the previously described search criteria, choosing clinical studies where the rates of true positive (TP), true negative (TN), false positive (FP) and false negative (FN) cases were available for constructing a 2×2 table. All references of these selected articles were also assessed for identifying other potentially includible studies. A pooled analysis based on the Mantel-Haenszel method was finally carried out, with the purpose of estimating the diagnostic sensitivity, specificity and accuracy (expressed as Summary Receiver Operating Characteristic Curve [SROC], agreement and Kappa statistics), with 95%CI and using a random effects model. Within studies heterogeneity was calculated using X^2^ test and I^2^ statistic. A second sub-analysis was conducted for assessing the diagnostic sensitivity of Ortho VITROS SARS-CoV-2 antigen test in studies that reported this information in specimens with high viral load. The statistical analysis was performed with Meta-DiSc 1.4 (Unit of Clinical Biostatistics team of the Ramón y Cajal Hospital, Madrid, Spain) [11].

**Table 1:** The search strategy summary.

Items	Specification
Date of Search	January 23, 2022
Databases and other<br>sources searched	Scopus, Medline<br>(PubMed interface)
Search terms used	»VITROS« AND »antigen« AND<br>»COVID-19« OR »SARS-CoV-2«<br>AND »immuno assay«
Timeframe	Up to January 23, 2022
Inclusion and<br>exclusion criteria	No date or language restrictions,<br>clinical studies where the rates of<br>true positive (TP), true negative<br>(TN), false positive (FP) and<br>false negative (FN) cases com-<br>paredto reference SARS-CoV-2<br>molecular biology techniques<br>were available for constructing a<br>2×2 table
Selection process	Conducted by G.L., verified by<rb>B.M.H.

This pooled analysis was conducted according to the Preferred Reporting Items for Systematic Reviews and Meta-Analyses (PRISMA Checklist available as Table 2), in accordance with the Declaration of Helsinki and within the terms of local legislation. No ethical committee approval was necessary, since this is a critical literature review.

**Table 2:** Preferred Reporting Items for Systematic Reviews and Meta-Analyses (PRISMA) Checklist. From: Page MJ, McKenzie JE, Bossuyt PM, Boutron I, Hoffmann TC, Mulrow CD, et al. The PRISMA 2020 statement: an updated guideline for reporting systematic reviews. BMJ 2021;372:n71. doi: 10.1136/bmj.n71

Section and Topic	Item<br>#	Checklist item	Location where<br>item is reported
TITLE			
Title	1	Identify the report as a systematic review.	Page 1
ABSTRACT			
Abstract	2	See the PRISMA 2020 for Abstracts checklist.	Page 2
INTRODUCTION			
Rationale	3	Describe the rationale for the review in the context of existing knowledge.	Page
Objectives	4	Provide an explicit statement of the objective(s) or question(s) the review addresses.	Page 4
METHODS			
Eligibility criteria	5	Specify the inclusion and exclusion criteria for the review and how studies were grouped for the syntheses.	Page 5 – Table 1
Information sources	6	Specify all databases, registers, websites, organisations, reference lists and other sources searched or consulted to identify<br>studies. Specify the date when each source was last searched or consulted.	Page 5 – Table 1
Search strategy	7	Present the full search strategies for all databases, registers and websites, including any filters and limits used.	Page 5–6
Selection process	8	Specify the methods used to decide whether a study met the inclusion criteria of the review, including how many reviewers<br>screened each record and each report retrieved, whether they worked independently, and if applicable, details of automation<br>tools used in the process.	Page 5–6
Data collection process	9	Specify the methods used to collect data from reports, including how many reviewers collected data from each report,<br>whether they worked independently, any processes for obtaining or confirming data from study investigators, and if<br>applicable, details of automation tools used in the process.	Page 5 –<br>Table 1
Data items	10a	List and define all outcomes for which data were sought. Specify whether all results that were compatible with each out-<br>comedomain in each study were sought (e.g. for all measures, time points, analyses), and if not, the methods used to<br>decide which results to collect.	Page 5–6
	10b	List and define all other variables for which data were sought (e.g. participant and intervention characteristics, funding<br>sources). Describe any assumptions made about any missing or unclear information.	Page 6
Study risk of bias<br>assessment	11	Specify the methods used to assess risk of bias in the included studies, including details of the tool(s) used, how many<br>reviewers assessed each study and whether they worked independently, and if applicable, details of automation tools used in<br>the process.	N/A
Effect measures	12	Specify for each outcome the effect measure(s) (e.g. risk ratio, mean difference) used in the synthesis or presentation of<br>results.	Page 6
Synthesis methods	13a	Describe the processes used to decide which studies were eligible for each synthesis (e.g. tabulating the study intervention<br>characteristics and comparing against the planned groups for each synthesis (item #5)).	Page 5-6 – Table 1
	13b	Describe any methods required to prepare the data for presentation or synthesis, such as handling of missing summary<br>statistics, or data conversions.	Page 5-6 – Table 1
	13c	Describe any methods used to tabulate or visually display results of individual studies and syntheses.	Page 5-6 – Table 1
	13d	Describe any methods used to synthesize results and provide a rationale for the choice(s). If meta-analysis was<br>performed, describe the model(s), method(s) to identify the presence and extent of statistical heterogeneity, and software<br>package(s) used.	Page 5-6
	13e	Describe any methods used to explore possible causes of heterogeneity among study results (e.g. subgroup analysis,<br>meta-regression).	Page 6
	13f	Describe any sensitivity analyses conducted to assess robustness of the synthesized results.	N/A
Reporting bias assessment	1	Describe any methods used to assess risk of bias due to missing results in a synthesis (arising from reporting biases).	N/A
Certainty assessment	15	Describe any methods used to assess certainty (or confidence) in the body of evidence for an outcome.	N/A
RESULTS			
Study selection	16a	Describe the results of the search and selection process, from the number of records identified in the search to the number<br>of studies included in the review, ideally using a flow diagram.	Page 6
	16b	Cite studies that might appear to meet the inclusion criteria, but which were excluded, and explain why they were excluded.	Page 6
Study characteristics	17	Cite each included study and present its characteristics.	Page 7 –<br>Table 2 & Table 3
Risk of bias in studies	18	Present assessments of risk of bias for each included study.	N/A
Results of individual<br>studies	19	For all outcomes, present, for each study: (a) summary statistics for each group (where appropriate) and (b) an effect estimate<br>and its precision (e.g. confidence/credible interval), ideally using structured tables or plots.	Page 7 –<br>Table 2 & Table 3
Results of syntheses	20a	For each synthesis, briefly summarise the characteristics and risk of bias among contributing studies.	N/A
	20b	Present results of all statistical syntheses conducted. If meta-analysis was done, present for each the summary estimate and<br>its precision (e.g. confidence/credible interval) and measures of statistical heterogeneity. If comparing groups, describe the<br>direction of the effect.	Page 7 –<br>Table 2 & Table 3 -<br>Figure 1 & Figure 2
	20c	Present results of all investigations of possible causes of heterogeneity among study results.	Figure 1 & Figure 2
	20d	Present results of all sensitivity analyses conducted to assess the robustness of the synthesized results.	Figure 1 & Figure 2
Reporting biases	21	Present assessments of risk of bias due to missing results (arising from reporting biases) for each synthesis assessed.	N/A
Certainty of evidence	22	Present assessments of certainty (or confidence) in the body of evidence for each outcome assessed.	Page 7
DISCUSSION			
Discussion	23a	Provide a general interpretation of the results in the context of other evidence.	Page 8
	23b	Discuss any limitations of the evidence included in the review.	Page 8
	23c	Discuss any limitations of the review processes used.	Page 8-9
	23d	Discuss implications of the results for practice, policy, and future research.	Page 8-9
OTHER INFORMATION			
Registration and protocol	24a	Provide registration information for the review, including register name and registration number, or state that the review was<br>not registered.	N/A
	24b	Indicate where the review protocol can be accessed, or state that a protocol was not prepared.	N/A
	24c	Describe and explain any amendments to information provided at registration or in the protocol.	N/A
Support	25	Describe sources of financial or non-financial support for the review, and the role of the funders or sponsors in the review.	Page 9
Competing interests	26	Declare any competing interests of review authors.	Page 10
Availability of data, code<br>and other materials	27	Report which of the following are publicly available and where they can be found: template data collection forms; data<br>extracted from included studies; data used for all analyses; analytic code; any other materials used in the review.	Upon request to<br>corr. author

## Results

The electronic search according to the predefined criteria allowed to identify 39 publications after eliminating duplicates among the two scientific databases. Thirty four of such document were not included since they did not present information on Ortho VITROS SARS-CoV-2 antigen test for diagnosing acute SARS-CoV-2 infection (n=23), were review articles (n=7), did not correlate test accuracy with a reference SARS-CoV-2 molecular diagnostic technique (n=3), or were a case report (n=1). Overall, 5 studies (n=2734 samples) could finally be included in our pooled analysis [12] [13] [14] [15] [16].

The main aspects of these five studies are presented in Table 3. Two studies were conducted in France, and one each in Canada, Japan and India. In four studies, nasopharyngeal swab only were used as reference diagnostic sample, whilst the remaining investigation used both oro- and naso-pharyngeal specimens [16]. All studies used the manufacturer's recommended diagnostic threshold of ≥1 S/C. The viral load in the study samples varied between 9 and 39 cycle threshold (Ct) values, with a cumulative sample size between 128 and 1727 specimens. In 4/5 included studies, a sub-analysis of diagnostic sensitivity in specimens with high-viral load was available (n=261), as shown in Table 4. In two of such studies the cut-off of high viral load was defined as Ct value ≤30, in one study as Ct value ≤25, and as ≥500 copies/reaction in the remaining study.

**Table 3:** Summary of studies that investigated the cumulative diagnostic performance of Ortho VITROS SARS-CoV-2 antigen chemiluminescent immunoassay for diagnosing acute SARS-CoV-2 infections. Ct, cycle threshold; NPS, nasopharyngeal swab; O-NPS, oro- and nasopharyngeal specimens; S/C, signal/cutoff ratio

Study	Country	Sample<br>matrix	Cut-off	Sample<br>size	Molecular assay (gene targets)	Viral load<br>(range)
Favresse et al. 2021	France	NPS	≥1 S/C	203	Roche LightMix Modular SARS-CoV<br>E-gene set (E)	13–38 Ct
Fourati et al. 2021	France	NPS	≥1 S/C	1727	ARGENE® SARS-COV-2 R-GENE (RdRp)	15–39 Ct
Levett et al. 2021	Canada	NPS	≥1 S/C	528	In house (E and RdRP)	14–37 Ct
Matsuzaki et al. 2021	Japan	NPS	≥1 S/C	128	In-house - Japan National Institute of Infectious<br>Diseases (NIID) method (N)	24–35 Ct
Paul et al. 2021	India	O-NPS	≥1 S/C	148	Altona RealStar SARS-CoV-2 RT-PCR Kit<br>(E and S)	9–33 Ct

**Table 4:** Summary of studies which investigated the diagnostic performance of Ortho VITROS SARS-CoV-2 antigen chemiluminescent immunoassay for diagnosing acute SARS-CoV-2 infections in oro- and nasopharyngeal samples with high viral load. Ct, cycle threshold; NPS, nasopharyngeal swab; O-NPS, oro- and nasopharyngeal specimens

Study	Country	Sample matrix	Sample size	Cut-off of high viral load
Favresse et al. 2021	France	NPS	77	≤30 Ct
Fourati et al. 2021	France	NPS	85	≤30 Ct
Matsuzaki et al. 2021	Japan	NPS	29	≥500 copies/reaction
Paul et al. 2021	India	O-NPS	70	≤25 Ct

**Figure 1:**
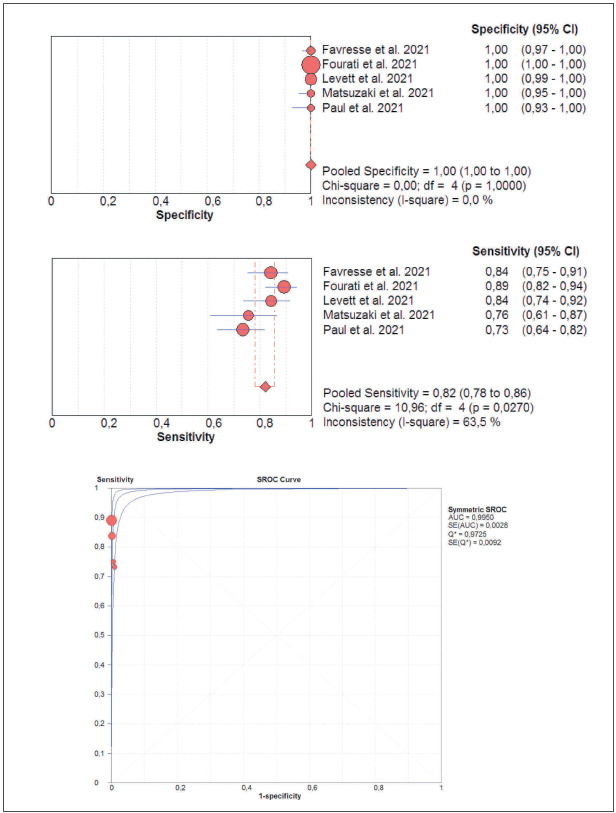
Cumulative diagnostic sensitivity and specificity with 95% confidence interval (95%CI) of Ortho VITROS SARS-CoV-2 antigen chemiluminescent immunoassay for diagnosing acute SARS-CoV-2 infections in oro- and nasopharyngeal samples.

**Figure 2:**
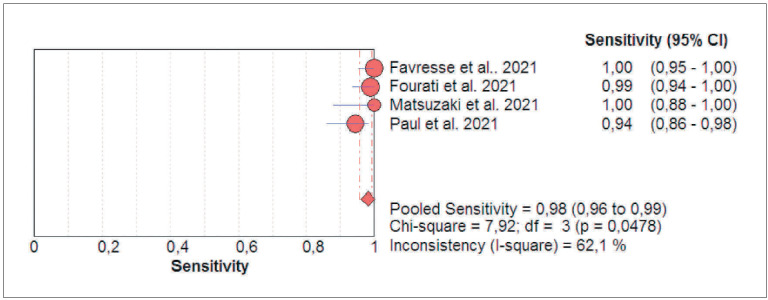
Diagnostic sensitivity of Ortho VITROS SARS-CoV-2 antigen chemiluminescent immunoassay for diagnosing acute SARS-CoV-2 infections in oro- and nasopharyngeal samples with high viral load.

The results of cumulative pooled analysis of diagnostic accuracy of Ortho VITROS SARS-CoV-2 antigen test for diagnosing acute SARS-CoV-2 infections are summarized in Figure 1. The pooled diagnostic sensitivity and specificity were 0.82 (95%CI, 0.78-0.86; I2, 63.5%) and 1.00 (95%CI, 1.00-1.00; I2, 0.0%), respectively, whilst the area under the SROC (AUC) was 0.995 (95%CI, 0.993-0.997), the diagnostic accuracy 97.2% (95%CI, 96.5-97.8%) with 0.89 (95%CI, 0.86-0.91) kappa statistics, thus reflecting an almost perfect agreement [17]. The pooled diagnostic sensitivity of Ortho VITROS SARS-CoV-2 antigen test in samples with high viral load is summarized in Figure 2, which allowed to calculate a pooled diagnostic sensitivity as high as 0.98 (95%CI, 0.96-0.99; I^2^, 62.1%).

## Discussion

Mass-scale SARS-CoV-2 testing and timely generation of test results are becoming compelling needs for providing optimal care and enabling an efficient contact tracing with new and highly transmissible variants such as Omicron (B.1.1.529) [18]. Nonetheless, the paramount number of diagnostic tests that would be needed to face the ongoing »fourth« wave of the COVID-19 pandemic are clearly overwhelming the molecular testing capacity of most laboratories worldwide. Although a NAAT remains the gold standard for diagnosing acute SARS-CoV-2 infections, the recent development of accurate, fast and high-throughput laboratory-based immunoassays is an important avenue of consideration for supporting high-volume and rapid diagnostic responses [19]. Among these methods, the Ortho VITROS SARS-CoV-2 antigen test represents a valuable alternative owing to the large diffusion of automated Ortho platforms in many clinical laboratories all around the world.

The results of this critical literature review of studies which evaluated this chemiluminescent immunoassay reveal that the pooled diagnostic accuracy is considerably high, as attested by the over 80% sensitivity, 100% specificity and 97.2% agreement with reference molecular techniques. These figures, which exactly match those claimed by the manufacturer (i.e., 80% and 100% percent positive and negative agreement, respectively), would make this method an important surrogate of molecular tests for purpose of mass (population) screening. This conclusion is further supported by the extremely high pooled diagnostic sensitivity (i.e., 98%) estimated in oro-and nasopharyngeal samples with high viral load. Notably, the cumulative diagnostic accuracy of this test seems to be even higher than that described for some similar immunoassays such as the Roche Elecsys SARS-CoV-2 antigen immunoassay (68% sensitivity, 99% specificity and 89% accuracy, respectively) [20], the DiaSorin LIAISON SARS-CoV-2 Ag test (31% sensitivity, 100% specificity and 50% accuracy, respectively) [21], and globally comparable to those of a high-sensitivity immunoassay such as Fujirebio Lumipulse SARS-CoV-2 Ag test (87% sensitivity, 97% specificity and 96% accuracy, respectively) [22].

It has now been clearly established that most SARS-CoV-2 infections are driven by the so-called »super-carries«, who incidentally become also »super-spreaders« in specific circumstances (i.e., limited or absence of physical protection during prolonged indoor contacts in schools, airports, stations, restaurants, bars and other public places, as well as during mass gatherings such as sports events, concerts, public manifestations and so forth) [23]. Several lines of evidence now attest that these »super-spreaders« would disproportionately concur to generate a huge number of SARS-CoV-2 transmissions and infections [24], in that less than 20% of infected individuals may be responsible for over 80% of all new cluster transmissions [25], and with such risk being magnified when infected people shed SARS-CoV-2 at very high viral loads and share many close contacts [26]. To this end, the availability of fast, accurate and high-throughput immunoassays capable to reliably identify subjects bearing high viral load not only may enable the rapid clinical management of those with suggestive symptoms needing timely and appropriate care, but would also allow to conduct extensive contact tracing, such that close contacts - especially those bearing high viral load - could be efficiently isolated, thus ultimately preventing or limiting further SARS-CoV-2 spread within the community.

## Conclusion

The results of this critical review and pooled analysis of the literature confirm that the automated and high-throughput Ortho VITROS SARS-CoV-2 antigen test represents a valuable surrogate of molecular testing for diagnosing acute SARS-CoV-2 infections, especially in subjects bearing a high viral load.

## Dodatak

### Research funding

None declared.

### Author contributions

All authors have accepted responsibility for the entire content of this manuscript and approved its submission.

### Informed consent

Not pertinent.

### Acknowledgments

None.

### Conflict of interest statement

All the authors declare that they have no conflict of interest in this work.
